# Emergency Medicine Research Priorities for Early Intervention for Substance Use Disorders

**DOI:** 10.5811/westjem.2019.1.39261

**Published:** 2019-02-19

**Authors:** Kathryn F. Hawk, Rachel L. Glick, Arthur R. Jey, Sydney Gaylor, Jamie Doucet, Michael P. Wilson, John S. Rozel

**Affiliations:** * Yale University, Department of Emergency Medicine, New Haven, Connecticut; † University of Michigan, Department of Psychiatry, Ann Arbor, Michigan; ‡ Sutter Medical Center, Department of Emergency Medicine, Sacramento, California; § University of California-San Diego, Department of Emergency Medicine, San Diego, California; ¶ Rosalind Franklin University, Chicago, Illinois; || University of Arkansas for Medical Sciences, Department of Emergency Medicine, Little Rock, Arkansas; # University of Pittsburgh, Department of Psychiatry, Pittsburgh, Pennsylvania

## Abstract

**Introduction:**

Patients with substance use disorders (SUDs) frequently seek emergency care, and the emergency department (ED) may be their only point of contact with the healthcare system. While the ED visit has been increasingly recognized as providing opportunity for interventions around substance use, many questions remain.

**Methods:**

In December 2016 the Coalition on Psychiatric Emergencies (CPE) convened the first Research Consensus Conference on Acute Mental Illness, which consisted of clinical researchers, clinicians from emergency medicine, emergency psychiatry, emergency psychology, representatives from governmental agencies and patient advocacy groups. Background literature review was conducted prior to the meeting, and questions were iteratively focused, revised, voted on and ranked by perceived importance using nominal group method.

**Results:**

The main goal of the SUD workgroup was to identify research priorities and develop a research agenda to improve the early identification of and management of emergency department (ED) patients with SUDs with the goal of improving outcomes. This article is the product of a breakout session on “Special Populations: Substance Use Disorder.” The workgroup identified with high consensus six research priorities for their importance related to the care of ED patients with SUDs in these overall domains: screening; ED interventions; the role of peer navigators; initiation of SUD management in the ED; specific patient populations that may impact the effectiveness of interventions including sociogenerational and cultural factors; and the management of the acutely intoxicated patient.

**Conclusion:**

Emergency providers are increasingly recognizing the important role of the ED in reducing adverse outcomes associated with untreated SUDs. Additional research is required to close identified knowledge gaps and improve care of ED patients with SUD.

## INTRODUCTION

In 2016, the National Survey on Drug Use and Health (NSDUH) demonstrated that only 2.3 million of the 20.5 million individuals with an identified need for treatment of a substance use disorder (SUD), had received care within the prior year.^1^ Nonetheless, patients with SUD frequently seek emergency care, making up half of the more than 4.9 million emergency department (ED) visits for drug-related complaints.^2^ Patients with unmet treatment needs are more likely to be hospitalized than those receiving treatment for a SUD, and substance use is associated with higher rates of unintentional injuries, motor vehicle collisions, interpersonal violence, human immunodeficiency virus infection, and intentional or accidental overdose.^3^–^6^ Increasingly, the ED visit has been identified as a unique opportunity for intervention and linkage to treatment for patients who are at risk for or who currently have SUDs, whether for tobacco, opioid or alcohol use.^7^–^10^

The ED may be the only point of contact with the healthcare system for some patients with SUDs. An ED visit for an acute injury, illness or overdose may provide a window of opportunity where patients are more receptive to education about and referral to treatment for SUD.^11^ Over the past decade, significant strides have been made in the field of ED-based identification, interventions and referrals for the treatment of SUD, but many questions remain. The goal of the SUD workgroup of the Coalition on Psychiatric Emergencies Research Consensus Conference on Acute Mental Illness was to identify research priorities and develop a research agenda to improve the early identification of and management of ED patients with SUDs with the goal of improving outcomes.

## METHODS

Please see the Executive Summary (Appendix) for full methods. Participants from a variety of disciplines – emergency medicine (EM), emergency psychiatry, emergency psychology, clinical research, governmental agencies, and patient advocacy groups – were invited to participate in a research consensus session held prior to a joint emergency-psychiatry conference (the 7^th^ Annual National Update on Behavioral Emergencies). Background literature reviews were performed prior to the in-person meeting. A total of 38 articles were circulated to the SUD group in advance. The working group initially identified three key areas: identification and diagnosis/screening; ED-based interventions; and linkages to the continuum of care. During the conference, the group spent time discussing research gaps related to addiction in the ED and identified 36 research topics. After spending time generating the initial 36 questions, the group re-reviewed them and held additional discussions to add clarity and intent; it then condensed the list to 24 questions.

A nominal group technique was employed to develop group consensus on the highest priority research gaps. Each member was given five points with which to vote for the questions they felt were most important. The questions were then ranked by the number of votes. The group identified six key research questions to guide ED-based interventions for SUD. Following the nominal technique, additional input was solicited from participants, questions were iteratively focused and revised, voted on, and then ranked by importance. Following the in-person session, the workgroup developed additional consensus by meeting electronically to further refine the final form of each question.

The working group focused on SUD was made up of seven people: one EM clinician researcher, one EM clinician, and two clinician psychiatrists; a non-physician student; a participant from a medical association, and an observer from industry. The average age of the participants was about 42 years old; four were females and three were males.

## RESULTS

During the consensus conference, research questions and topics were sequentially proposed by individual members of the workgroup, and were transcribed into a large working board visible to all group members in real time. Workgroup members proposed research topics individually and in a sequential fashion, for a total of 36 research topics. Topics were discussed, grouped, voted on, and prioritized using the nominal group technique.

## DISCUSSION

The workgroup identified six questions as the highest priority areas for early identification of SUDs in the ED. (Additional questions and discussion, organized by topic, are also included in Table 1 and Table 2.)

### Screening

Based on a robust literature search, the United States Preventive Services Task Force (USPSTF) and the American College of Emergency Physicians (ACEP) recommend screening and brief intervention for alcohol use disorders in primary care and ED settings.^11^–^13^ The role of universal screening for illicit drug use, either in the primary care or ED setting, is less clear. Although evidence is lacking, increased rates of illicit drug use among ED patients, recent increases in opioid-associated mortality, and recent ED-based studies showing improved outcomes after ED intervention, provide a basis for the role of ED screening for SUDs.^8^,^12^,^14^–^16^

While ED-based research studies focused on SUDs have used screening to identify potential study subjects, little is known about either the impact or the most effective implementation of ED-wide screening procedures in the day-to-day functioning of an ED. Multiple studies have adapted, developed and piloted a variety of screening tools for SUDs using tablet- and kiosk-based platforms, but consensus on the most effective implementation of ED-based screening algorithms outside of a research study has not been reached.^2^,^7^,^17^,^18^ Several EDs have implemented Screening, Brief Intervention and Referral Treatment (SBIRT) programs including use of health promotion advocates such as in Project Alcohol and Substance Abuse Services, Education, and Referral to Treatment (ASSERT), or training ED residents and faculty as part of Substance Abuse and Mental Health Services Administration’s (SAMSHA) SBIRT training grants as best practice.^19^

Although several of these programs are of long standing and have linked thousands of ED patients to SUD care, the most effective and efficient way to screen in diverse ED settings remains unclear.^12^ Importantly, the logistics of who administers the screen and how it is performed (e.g., triage nurse; tablet-based or self-administered; emergency provider) will influence the overall acceptability of the process to the patient, the sensitivity to detect SUDs, the integration of the process into the ED workflow, and the overall sustainability. The most efficient and effective approach to screening for SUDs will likely vary based on patient population, geography, ED volume, community resources, ED staffing and academic vs community hospital settings, and may vary across cultural and generational patients within ED populations. Increasingly, the ED has been recognized as an important venue to identify and engage patients with SUDs.^13^

### Intervention

Although significant strides have been made in improving outcomes for ED patients with risky alcohol use, uncertainly surrounding the most effective interventions to reduce illicit drug use persist. A fairly robust literature exists supporting the implementation of SBIRT for alcohol use disorders in primary care settings^11^,^20^ although mixed results have been seen in the ED.^7^,^21^,^22^ Brief interventions incorporate principles of motivational interviewing, an evidence-based counseling technique that uses empathy, positive framing, reflective listening, and gentle education to enhance motivation to reduce risky behaviors.^10^,^23^ Brief interventions for patients with at-risk or hazardous drinking usually focus on reducing use, while the focus for patients with dependence is on enhancing motivation to accept a referral to formal treatment.^13^–^15^ Some ED-based studies have shown success in reducing alcohol consumption, episodes of binge drinking and episodes of driving after drinking in harmful and hazardous alcohol drinkers, although other studies have been less encouraging, with no persistent effect at one year.^7^,^22^,^24^,^25^

ED-based brief interventions for drug use have been less promising. The Screening, Motivational Assessment, Referral, and Treatment in Emergency Departments (SMART-ED) Clinical Trials Network Study across six academic EDs did not detect differences in drug use at any point in time.^16^ Additionally, a single, large, randomized control trial (RCT) found that a brief motivational intervention for patients with alcohol or drug use disorders did not improve attendance at post-ED intervention over a case management intervention.^16^,^18^,^19^ However, there were several methodological issues with these studies, and it is likely that one intervention may not be effective for all types of drugs at all levels of severity.

More recently, ED-based interventions specific to patients with opioid use disorders (OUD) have shown more promise. One pilot RCT of ED patients with non-medical opioid use found a significant reduction in overdose-risk behaviors and a reduction in non-medical opioid use at six months after an ED-based motivational interview intervention compared to usual care.^9^ In one single ED-based RCT, patients with opioid dependence who received a brief intervention and ED-initiation of buprenorphine were significantly more likely to be engaged in treatment for OUD at 30 days (78% vs 37%) and had fewer days of opioid use than the standard referral to treatment group.^20^ This study, augmented by the persistent rapid rise of opioid-associated fatalities, has prompted a number of EDs across the country to develop programs initiating treatment with buprenorphine for OUD in the ED, although many questions remain about how to optimize implementation, patient selection, models of linkage and induction/dosing algorithms to maximize safe and effective linkage to treatment.^21^,^22^ Studies are needed to optimize these and other strategies to enhance the success of ED-initiated buprenorphine, and to better characterize patient and provider facilitators and barriers to the implementation of this intervention.

In the general population in the Western world, approximately 10% of women and 20% of men will have an alcohol use disorder (AUD).^23^ About 50% of individuals with AUD are expected to have withdrawal symptoms with reduction or cessation of alcohol use, and 3–5% will have severe complications of withdrawal including seizures or delirium.^23^ That said, ED clinicians routinely care for those with the highest risk of complicated withdrawal. General consensus and non-ED based literature suggest patients with mild to moderate AUD may be appropriate for outpatient management with or without oral benzodiazepines.^29^,^30^ However, there is a paucity of prospective, ED-based studies to provide guidance for the ED population.

Patients at high risk for severe withdrawal and therefore generally inappropriate for outpatient management, include those with a history of alcohol withdrawal seizure or delirium, psychiatric or medical co-morbidities, or patients who receive multiple doses of benzodiazepines without significant reduction in Clinical Institute Withdrawal Assessment scale.^24^ Clinical decisions regarding the disposition of patients at risk for or with symptoms of alcohol withdrawal are challenging given the dearth of prospective, evidence-based ED studies to guide the risk-benefit analysis of discharging the patient who is at risk for moderate to severe alcohol withdrawal. Moreover, although multiple outpatient regimens for the treatment of alcohol withdrawal symptoms have been described, no clear evidence exists for the most appropriate medication type and dosing schedule.^23^,^25^,^26^

### Patient Population Factors

Little is known about the role of sociocultural and generational factors in the acceptability, accessibility, and benefit of ED-based initiatives to reduce harmful substance use and provide linkage to treatment for SUD. Many novel interventions rely on relatively new mobile health and other technology, including smartphone, text messaging and videoconferencing-based interventions, or wearable biosensors, which many be more appealing to younger patients, but create an additional barriers for identifying or intervening in substance use for populations with less intrinsic exposure to technology because of cultural factors or age.^27^–^29^ Although intervention developers may be specifically targeting younger patients, cultural and generational factors should be considered in the development and implementation of ED-based initiatives given the pervasive distribution of SUDs across all demographics.^1^–^3^

### Initial Substance Use Disorder Management

Intoxicated patients present unique challenges to the emergency physician. They can be agitated and disruptive.^30^ Patients present with alcohol intoxication alone or in combination with other drugs, but an increasing number of visits are due to stimulants, novel psychoactive substances (NPS) and designer drugs.^31^ Literature on management of alcohol intoxication exists but is built on consensus and our limited knowledge of treating agitation in general. Little research has been done on the best management for stimulants and newer substances and the current literature consists mainly of descriptive small series, case reports, or surveys of clinicians’ experiences.^3^

Of the more than 4.5 million ED visits in 2009 for drug-related causes,^34^ 32% involved alcohol use alone or in combination with other drugs. Nearly 94,000 visits were for stimulants and over 400,000 were for cocaine, while fewer were for phencyclidine, gamma hydroxybutyrate, and ecstasy.^35^ While it is not clear how many of these visits were for substance-related intoxication as opposed to withdrawal, drug seeking or other reasons, it is clear that the intoxicated patient presents unique challenges to the ED treatment team. Furthermore, the burden of caring for patients with acute, alcohol-related visits more than doubled between 2001 and 2011 reflecting an increased number of visits, longer length of stay, and more intensive use of diagnostic services.^36^

When a patient presents with suspected drug intoxication and is sleepy or sedated, management is straightforward and supportive until the substance clears and the patient awakens. When a patient is agitated, disruptive, and not cooperative, management is more difficult. Management of agitation in general is not well studied,^37^ and it is not surprising that our understanding of the best approach to managing the patient who is agitated because of intoxication is limited and based more on retrospective reviews, anecdotal information, and expert consensus.^38^,^39^ Experts recommend identification of drugs/alcohol as cause of agitation as a first step, followed by verbal de-escalation and medications as necessary, but the research to back this approach is lacking.^40^–^41^

Another issue to consider when treating intoxicated patients in the emergency setting is the value of laboratory testing such as drug screens and blood alcohol levels. Available toxicology screens often miss substances, and patient history may be more helpful than expensive diagnostic tests except in situations where patients are obtunded or otherwise unable to provide a history.^42^–^43^ As clinical intoxication frequently does not align with blood alcohol levels, questions frequently arise in determining patient ability to make medical decisions, including the ability to refuse medical care or, depending on the ED setting, when patients are appropriate for psychiatric evaluation.^44^–^46^ No clear evidence-based consensus currently exists on the best practices for medical workup prior to psychiatric evaluation.

Substance use is a well-known risk factor for suicide, and a large percentage of individuals who die by suicide are intoxicated at the time of their deaths.^47^ One challenge is how to best assess risk of suicide in the patient who presents to the ED with suicidal statements when intoxicated but later recants when sober saying they either “just said those things” because they were intoxicated or denying any memory of making suicidal statements or having suicidal thoughts. Persistent knowledge gaps exist around best practices for this ED population.

### Peer Mentors

Peer mentors, people with the lived experience of recovery from addiction and mental illness, are becoming increasingly common in the healthcare landscape.^48^ Peer mentors have been identified as a potential bridge to treatment for ED patients after non-fatal opioid overdose, although the impact of this approach on outcomes is unclear.^49^ Early indicators suggested that using peer mentors and peer-led programs can be a helpful diversion for people with addiction and mental health emergencies.^50^–^51^ Larger studies have shown limited benefit from peer interventions, often due to inconsistent program fidelity and heterogeneous approaches.^52^ Emerging efforts to create fidelity models are promising.^53^ However, interventions need to be evidenced based and administered by individuals adhering to critical actions with routine fidelity checks and supervision. Additional research to explore the impact of a potentially important and effective way to support and engage people with addiction emergencies, including after opioid overdose, who require linkage to early recovery resources are needed.

## LIMITATIONS

There are several limitations to this study. First, this was not a structured review of literature but rather the outcome of an expert consensus group meeting that was held in 2016. By the time of this paper’s publication, it is possible that studies may have been conducted that answer or speak to some of the highlighted questions raised. Second, the group focus was narrowed to the early identification and management of patients presenting to the ED with SUDs, drugs and alcohol. Although we recognize the impact on tobacco use disorder and other medical and psychiatric comorbidities, given our limited time, we limited the scope of our work to the care and management of SUDs in the acute care settings and thus we did not specifically discuss tobacco or include focus on the management of other comorbidities. As with many in-person consensus conferences, participation is limited to those who were able to travel and attend in person; had all of the original invitees or others with valuable experience been able to attend, the findings may have been different. Nonetheless, we have highlighted a number of priority areas in which additional research is clearly needed and that can guide ongoing research as we work to improve outcomes of ED patients with SUDs.

## CONCLUSION

Emergency providers are increasingly recognizing the important public health role that EDs can play to reduce adverse outcomes associate with undiagnosed and untreated substance use disorder. Much like the ED is the “front door” for hospital admission, it is also a portal to the community to identify patients with SUDs and to deliver interventions to improve outcomes.

## Supplementary Material



## Figures and Tables

**Table 1:** Key research questions to guide emergency department-based interventions for substance use disorders.

Question 1	What are the most effective, efficient and appropriate ways to screen for SUD in the ED?
Question 2	What are the most effective ED-based interventions for SUD?
Question 3	What is the role for initiation and management of SUD treatment and detoxification in the ED?
Question 4	What is the role of sociocultural and generational factors in acceptability, accessibility, and benefit of ED-based initiatives?
Question 5	What are the best practices for the evaluation and management of the acutely intoxicated patient?
Question 6	What role can peer mentors, or patient navigators, play in improving patient outcomes?

*SUD,* substance use disorder; *ED*, emergency department.

**Table 2:** Key research questions to guide efforts for improved care of individuals with substance use disorders.

Topic area 1: What are the most effective, efficient and appropriate ways to screen for SUD in the ED? What is the best approach for sensitively and effectively screening for drug and alcohol use in the ED? How effective are current screening tools in different populations and do results vary with patient characteristics/identity: generational (i.e., millennial vs geriatric), gender, religious, cultural factors? What is the most cost-effective way to implement high-quality ED-based screening for SUD ? What is the role for SUD screening in ED triage? For universal screening?
Topic area 2: What are the most effective ED-based interventions for SUD? Which ED-based interventions can reduce cost, reduce mortality and increase treatment adherence? Do harm reduction initiatives (i.e., overdose prevention education, naloxone distribution) improve outcomes? To which types of treatment/services should ED patients with SUD be referred?
Topic area 3: What is the role for initiation and management of SUD treatment and detoxification in the ED? Who is appropriate for ED-initiated outpatient treatment of alcohol withdrawal? Is there a need for development of a validated ED-based protocol for initiating outpatient treatment of alcohol withdrawal? Is there a need for development of a validated ED-based protocol for initiating buprenorphine for the treatment of OUD, including who is most likely to benefit and when?
Topic area 4: What is the role of sociocultural and generational factors in acceptability, accessibility, and benefit if ED-based initiatives?
Topic area 5: What are the best practices for the evaluation and management of the acutely intoxicated patient? Do better evaluation, diagnosis and treatment of agitation of patients with acute intoxication of traditional drugs of abuse as well as newly emerging novel psychoactive substances (NPS) currently exist? Are there clinical guidelines for management of acute stimulant intoxication? What is the appropriate role of drug/toxicology screens in the ED? Is there evidence based-criteria for medical workup prior to psychiatric evaluation?
Topic area 6: What role can peer mentors, or patient navigators, play in improving patient outcomes? What role can peer mentors, or patient navigators, play in improving patient outcomes?

*SUD,* substance use disorder; *ED*, emergency department; *NPS*, novel psychoactive substances; *OUD*, opioid use disorder.
