# Supplementary material for: Emergency Medicine Research Priorities for Early Intervention for Substance Use Disorders
Source: West J Emerg Med. 2019 Feb 19;20(2):386–92. doi: 10.5811/westjem.2019.1.39261 (PMC6404722; doi:10.5811/westjem.2019.1.39261)
Supplement: Supplementary file 1 [file wjem-20-386-s001.docx]

# METHODS

**The Coalition on Psychiatric Emergencies (CPE)**

The CPE includes over a dozen professional organizations, patient advocacy groups, clinicians, and systems of care, all of which have an interest in behavioral emergencies.^18^ The steering committee at the time of the conference consisted of representatives from the following organizations: the American College of Emergency Physicians, the American Association for Emergency Psychiatry, the Depression and Bipolar Support Alliance, the National Alliance on Mental Illness, and the Emergency Nurses Association. Collectively, these organizations represent patients, patient advocacy groups, social workers, emergency physicians, psychiatrists, nursing, and clinical researchers. The steering committee was responsible for planning the conference, inviting participants and stakeholders, preliminary identification of domains for exploration, and determining methodology.

**Identification of Relevant Stakeholders**

Identification of relevant stakeholders (ie, conference participants) was accomplished primarily by a web search for publications in each particular domain. As this method of identification would be expected to weight the participant list most heavily toward researchers, non-clinicians with relevant interest and expertise were additionally identified by organizations on the CPE steering committee. As the Research Consensus Conference on Acute Mental Illness is the first-ever conference on behavioral emergencies to include non-clinicians, these participants were sorted into workgroups either by their work on behalf of patients or by national presentations.

**Conference Methodology**

The overall hypothesis of the conference was that, similar to other critical conditions in the emergency department, early treatment may positively affect outcomes for patients with mental health crises.^19^ Consistent with this hypothesis, the CPE steering committee identified four critical domains that formed the subsections of the consensus conference: geriatric behavioral health emergencies; suicidality and acute depression; substance use disorders; and acute psychosis. As in previous conferences of these types, the four domains were chosen a priori based on their importance to providers currently caring for patients with behavioral emergencies, and also to avoid duplication of previous consensus-conference efforts.^1, 17^ Once assembled, working groups in each domain were free to identify any relevant questions within their respective domains.

As patient advocacy on behalf of mental health patients often overlaps multiple, traditionally-identified research areas, participants from each group were able to provide feedback and comments on the priorities identified by other working groups both during the conference and after. Each workgroup appointed a moderator who conducted the consensus building during the conference and a group leader who identified all relevant articles prior to meeting in person. Each participant was provided with an electronic version of these articles prior to the conference.

Consensus building on research questions within each domain was accomplished by use of the nominal group technique.^20 21^ The nominal-group technique is a four-step process in which participants are invited to identify ideas and raise exploratory questions, record these ideas, discuss them freely, iteratively focus and revise them, and then vote on relative importance. Participants work independently but in the presence of one another. This method was chosen as it has the advantage of preventing any particular expert or advocate from dominating the conversation or influencing the voting.

Specific research ideas and questions were voted on using the dot method. Questions that received more votes were deemed to be more important, and thus were ranked more highly within each domain. As research on behavioral emergency questions are of importance to industry, representatives of pharmaceutical companies were permitted to attend in an observational role. However, representatives were not allowed to vote on the final wording or rank order importance of any question.

Via an iterative process, areas of priority selected by the dot method were then categorized into groups by participants of each working group. Groups were then free to revise and re-rank these questions as needed, both during the conference and after. No limits were set on the number of questions chosen by each group.
